# Combined inhibition of MEK and mTOR has a synergic effect on angiosarcoma tumorgrafts

**DOI:** 10.3892/ijo.2015.2989

**Published:** 2015-05-06

**Authors:** NICHOLAS J. ANDERSEN, ELISSA B. BOGUSLAWSKI, CYNTHIA Y. KUK, CHRISTOPHER M. CHAMBERS, NICHOLAS S. DUESBERY

**Affiliations:** 1 Laboratory of Cancer and Developmental Cell Biology, Van Andel Research Institute, Grand Rapids, MI 49503, USA; 2 Frederik Meijer Heart and Vascular Institute, Spectrum Health Hospital, Grand Rapids, MI 49503, USA

**Keywords:** angiosarcoma, sarcoma, MEK, MAPK, ERK, tumorgraft, cancer, combination therapy

## Abstract

Angiosarcoma (AS) is a rare neoplasm of endothelial origin that has limited treatment options and poor five-year survival. Using tumorgraft models, we previously showed that AS is sensitive to small-molecule inhibitors that target mitogen-activated/extracellular-signal-regulated protein kinase kinases 1 and 2 (MEK). The objective of this study was to identify drugs that combine with MEK inhibitors to more effectively inhibit AS growth. We examined the *in vitro* synergy between the MEK inhibitor PD0325901 and inhibitors of eleven common cancer pathways in melanoma cell lines and canine angiosarcoma cell isolates. Combination indices were calculated using the Chou-Talalay method. Optimized combination therapies were evaluated *in vivo* for toxicity and efficacy using canine angiosarcoma tumorgrafts. Among the drugs we tested, rapamycin stood out because it showed strong synergy with PD0325901 at nanomolar concentrations. We observed that angiosarcomas are insensitive to mTOR inhibition. However, treatment with nanomolar levels of mTOR inhibitor renders these cells as sensitive to MEK inhibition as a melanoma cell line with mutant BRAF. Similar results were observed in B-Raf wild-type melanoma cells as well as *in vivo*, where treatment of canine AS tumorgrafts with MEK and mTOR inhibitors was more effective than monotherapy. Our data show that a low dose of an mTOR inhibitor can dramatically enhance angiosarcoma and melanoma response to MEK inhibition, potentially widening the field of applications for MEK-targeted therapy.

## Introduction

Angiosarcoma (AS) is a rare and aggressive malignancy of the endothelium (reviewed in refs. 1,2). Angiosarcoma has no single identified cause. Rather, mutations in several different genes have been reported. Most recently, Bejhati *et al* reported that mutations in PTPRB and PLCG1 were detected in 10/39 and 3/34 tumors, respectively (3). In addition, constitutive activation of KRAS-2 (4–6) and VEGF receptor 2 (7) have been documented. Both of these signal through the mitogen-activated protein/extracellular-regulated kinase (MAPK/ERK) signaling pathway. Consistent with this, we have reported that AS shows focal to widespread ERK activity and expresses ERK-responsive genes (8). Furthermore, canine angiosarcoma tumorgrafts are sensitive to inhibitors that target MAPK/ERK kinase (MEK), the upstream activator of ERK (8). These data indicate the MEK/ERK pathway plays a central role in AS tumor growth.

MEK 1 and 2 are kinases that drive diverse basic biological processes such as cellular proliferation and cellular survival. Aberrant activation of these kinases has been linked with developmental syndromes and to as many as one-third of all cancers (reviewed in refs. 9,10). While MEK activation is predominately associated with melanoma (11), MEK dependency has been documented in a variety of other cancers, including osteosarcoma (12), Ewing sarcoma (13), fibrosarcoma (10,14), and Kaposi sarcoma (15). Thus, the MEK/ERK pathway is a therapeutic target with a broad spectrum of applications.

Despite the well-documented role of MEK signaling in cancer, MEK inhibitors historically have had limited utility in the clinic. The MEK1/2 inhibitor CI-1040 showed poor efficacy in Phase II study (16). PD0325901, a CI-1040 derivative, also showed poor tumor response in Phase II clinical study (17), and dose increases were limited by neurological and ocular toxicities (18). Currently, trametinib is the only FDA-approved MEK inhibitor for advanced melanoma. Even with this success, trametinib has failed to show additional benefit in patients who had been treated with BRAF inhibitors (19). Additional therapeutic strategies are needed to overcome dose-response and resistance mechanisms.

Combinations of multiple drugs having different mechanisms of action have been used effectively to treat diseases such as HIV, cancer, and bacterial infections (20–22), but the combined effects of drugs are not easily predicted. The combination often acts like a third drug with effects that are distinct from those of the original drugs (23). Moreover, the interaction of the combined drugs can be influenced by the cellular or genetic context in which they meet. Such interactions between drugs can promote greater selectivity, efficacy, lower toxicity, and delayed resistance, but they can also be antagonistic or promote greater toxicity. We and others have observed that one ratio of combined drugs may have a synergic effect but a different ratio of the same drugs may act in an antagonistic fashion (23). Thus, designing a combinatorial therapy first requires a rigorous *in vitro* evaluation to determine the optimal ratios and doses to elicit the greatest response. Since their interaction can be influenced by the cellular or genetic context, an *in vitro* evaluation must be performed for each tumor type tested. Finally, because strategies that are additive or synergic for tumor response may instead be more toxic, any new combination therapy requires an equally rigorous *in vivo* evaluation of toxicity and efficacy.

Herein we report our efforts to identify drugs that synergize with the MEK1/2 inhibitor PD0325901 in order to design a more effective therapy for angiosarcoma. Drugs were selected based on their ability to inhibit 11 of the conserved cancer pathways (24). The goal of these tests was to identify the optimal drug combination, i.e., the combination showing the greatest additive or synergic interaction with effective inhibition of cell viability at the lowest concentration. Using a systematic approach, we have discovered that angiosarcomas are insensitive to mTOR inhibition. However, treatment with nanomolar levels of an mTOR inhibitor renders these cells as sensitive to MEK inhibition as melanoma with mutant BRAF. Similar results were observed in B-Raf wild-type melanoma and *in vivo*, where treatment of canine AS (cAS) tumorgrafts with MEK and mTOR inhibitors is more effective than monotherapy. Our results show that a low dose of an mTOR inhibitor can dramatically enhance the response to MEK inhibition and thus may widen the field of applications for MEK-targeted therapy.

## Materials and methods

### Cell culture

cAS primary isolate VCT115 was grown as previously described (8). VCT220, VCT345, and VCT511 were isolated from cAS tumor samples as previously described (8) and were grown in DMEM containing 10% heat-inactivated fetal bovine serum (FBS; Life Technologies, Carlsbad, CA, USA) and 1% penicillin/1% streptomycin (Life Technologies). VCT261e was isolated from cAS tumor as previously described (8) and grown in EGM (Lonza, Basel, Switzerland) supplemented with EGM2 SingleQuots (Lonza). The melanoma-derived SK-MEL28 cells were grown as described, and WM-3211 (Coriell Institute, Camden, NJ, USA) cells were grown as previously described (25,26).

### In vitro combination index studies

PD0325901, sorafenib tosylate, dasatinib (LC Laboratories, Woburn, MA, USA), rapamycin doxorubicin, Nutlin-3a, SGX-523 (Selleck, Houston, TX, USA), and KT5270 (Santa Cruz Biotechnology, Dallas, TX, USA) were prepared in DMSO (Sigma-Aldrich, St. Louis, MO, USA). Cells were seeded into a 96-well plate using an epMotion 5075 pipetting system (Eppendorf, Hamburg, Germany). Treatments began when cells reached 30% confluency. Cells were treated for 72 h. Cell viability was measured using the CellTiter 96 Aqueous Non-Radioactive Cell Proliferation Assay (Promega, Madison, WI, USA) according to the manufacturer’s instructions. Assays were measured using a Benchmark Plus microplate spectrophotometer (Bio-Rad, Hercules, CA, USA) at 490 and 700 nm reference wavelengths. Cell viability was performed twice in duplicate wells. Treated wells were normalized to non-treated wells and then were normalized to DMSO-treated plates. The concentration of compound required to cause 50% inhibition of cell viability (IC_50_) was calculated (23). A combinatorial index was calculated following Chou and Talalay (23). If a drug combination resulting in synergy (CI<1) that combination was repeated for a total of three separate experiments in duplicate wells. The IC_50_ and CI were then calculated as previously described (23).

### Western blot analysis

The cAS primary SK-MEL28 and WM-3211 cells were seeded in the appropriate medium and were incubated overnight. Cells were grown to 30% confluency, then treated with a drug combination near the IC_60_ of the optimal drug molar ratio for 72 h. Total cell lysates were collected in RIPA buffer [50 mM Tris-HCl, 150 mM NaCl, 1 mM EDTA, 1 mM EGTA, 2 mM Na_3_VO_4_, 20 mM sodium pyrophosphate, 1% Triton X-100, 1% sodium deoxycholate, and 0.1% SDS, with Complete EDTA-free Protease Tablets (Roche Corp., Palo Alto, CA, USA)] and were sonicated three times using a Misonix Sonicator 3000 (Farmingdale, NY, USA). Protein concentrations were determined using the BCA Protein Assay kit (Pierce, Rockford, IL, USA). Cellular lysates were resolved in Novex Pre-Cast Tris-glycine gels (Life Technologies) and then transferred onto polyvinylidene fluoride (PVDF) membranes (Millipore, Billerica, CA, USA). Membranes were blocked with 10% non-fat milk and then incubated with antibodies against phospho-ERK (Thr202/Tyr204) (E10; Cell Signaling, Danvers, MA, USA), ERK (Cell Signaling), α-tubulin (Sigma-Aldrich), phospho-S6 (S235/236) (2F9; Cell Signaling) phospho-4E-BP1 (S65) (Cell Signaling), 4E-BP1 (53H11, Cell Signaling), and Bim (C34C5, Cell Signaling). The membranes were washed three times with TBST (50 mM Tris, 150 mM NaCl, and 0.1% Tween-20) and then incubated with the appropriate HRP-conjugated secondary antibodies (KPL, Gaithersburg, MD, USA) overnight at 4°C. Washed blots were incubated with Super Signal West Pico Chemiluminescent Substrate (Fisher Scientific, Pittsburgh, PA, USA) and were exposed to Hyblot CL film (Denville Scientific, South Plainfield, NJ, USA). The film was processed in an X-OMAT 2000A Processor (Kodak, Rochester, NY, USA).

### In vivo combination studies

Mice were bred and maintained according to established guidelines and a protocol approved by VARI’s Institutional Animal Care and Use Committee. For the toxicity studies, athymic nudes were treated at a 4:1 molar ratio PD0325901:temsirolimus (Selleck) (with a range of 4.38/2.35 to 3.5/1.87 mg/kg) for two weeks. PD0325901 was administered daily by oral gavage in 100 μl of 0.5% hydroxylpropyl methylcellulose plus 0.2% Tween-80 (27). Temsirolimus was administered i.p. in 200 μl using a 5 days on, two days off schedule. A 50 mg/ml stock of temsirolimus was prepared in 100% ethanol. On day of injection, it was diluted with 5% Tween-80, 5% polyethyleneglycol-400 for a final concentration of 0.4% ethanol as previously described (28). Treatment was initiated when tumors reached 50–100 mm^3^. Tumors were randomly selected into five different treatment arms: PD0325901 (3.5 mg/kg) and temsirolimus (1.87 mg/kg), PD0325901 single therapy (3.5 mg/kg), temsirolimus single therapy (1.87 mg/kg), vehicle, and non-treated. Monotherapy animals were given the corresponding vehicle on the appropriate schedule. Vehicle animals were given both vehicles on the appropriate schedule. Mice were weighed and monitored three times a week. Mice were sacrificed when tumors reached 1000 mm^3^ or treatment day 38, which ever came first, and terminal bleeds were collected for biochemical serum analysis (Abaxis VetScan Blood Chemistry Analyzer, Abaxis, Union City, CA, USA). cAS cardiac-derived tumorgrafts were characterized and implanted into athymic nude mice as previously described (8).

### Immunohistochemistry

Formalin-fixed, paraffin-embedded tumors were sectioned and immunostained with optimized standard protocols using a Ventana Discovery XL instrument (Ventana Medical Systems, Tucson, AZ, USA) and antibodies against phospho-ERK (Thr202/Tyr204) (20G11; Cell Signaling), CD31/PECAM-1 (Lab Vision, Kalamazoo, MI, USA), phospho-S6 (S235/236) (2F9; Cell Signaling), and Ki67 (ab833; ABCAM, Cambridge, MA, USA). Slides were incubated with HRP-conjugated anti-rabbit IgG secondary antibody (Ventana Medical Systems) and developed with 3-3′-diaminobenzidine (DAB) chromagen substrate. Images were acquired using a Nikon E800 Epifluorescent microscope equipped with a Spot RT3 CCD camera (Diagnostic Instruments, Sterling Heights, MI, USA) and Spot Advanced software.

## Results

### Synergism in vitro

**Figure 1:**
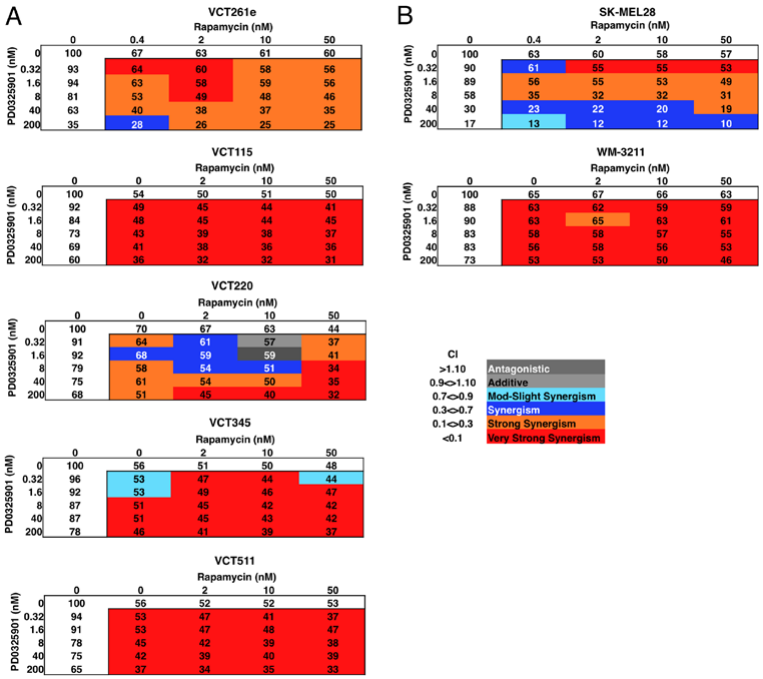
Combined treatment with PD0325901 and rapamycin has a synergic effect on (A) angiosarcoma and (B) melanoma viability. Average cell viability (%) and corresponding CI (color code) for PD0325901 in combination with rapamycin. The values shown are for three replicates of three parallel experiments in which cells were treated for 72 h.

We have published data showing that human and canine angiosarcomas express focal to widespread active ERK and are sensitive to MEK inhibition (8). In this follow-up study, we wanted to identify drugs or compounds that synergize with the MEK inhibitor PD0325901. We treated five canine angiosarcoma cell isolates with different ratios of the drugs and evaluated the effect on cell viability after a 72-h treatment (Fig. 1A). Among the drugs we tested, mTOR inhibitor rapamycin showed the strongest synergy with PD0325901 even at subnanomolar concentrations (Table I, and data not shown). PD0325901 plus rapamycin had the greatest synergy (CI≤0.08). 4 of the 5 angiosarcoma primary cell isolates had an optimal 4:1 molar ratio of PD0325901:rapamycin and the 5th had a 4:5 ratio (Table I, and data not shown).

To determine whether this response was unique to angiosarcoma or was a consequence of their MEK dependency, we performed the same experiments in melanoma-derived cell lines that were MEK-dependent (SK-MEL28, which has a BRAF V600E mutation) and MEK-independent (WM-3211, which is BRAF wild-type but contains a c-kit L576P mutation) (Fig. 1B). SK-MEL28 were sensitive to MEK and mTOR inhibition, but showed greater sensitivity to combined inhibition. In contrast, the WM-3211 line was insensitive to either MEK or mTOR inhibition but showed enhanced sensitivity to combination therapy (Table I). Thus, treatment with an mTOR inhibitor renders angiosarcomas as sensitive to MEK inhibition as melanomas having mutant BRAF, and it renders MEK inhibitor-resistant cells sensitive to MEK inhibition.

### Increased inhibition of canine AS tumorgrafts using combined MEK and mTOR inhibitors

*In vitro* combination matrices detailed the PD0325901 and rapamycin dual treatment at 4:1 molar ratio was the most efficacious. This dual treatment regimen was then examined *in vivo* on patient derived xenografts. Before the drug study was initiated, *in vivo* toxicity testing was performed to determine whether the combined therapy was safe in mice. For these studies we used the mTOR inhibitor temsirolimus, which is a pro-drug that is metabolized to yield rapamycin *in vivo* (29). Temsirolimus was used because, compared with rapamycin, it has a more favorable pharmacokinetic profile and greater solubility in water (30). Using 4:1 combinations of PD0325901 plus temsirolimus, over a two-week period we observed significant (>10%) weight loss, elevated serum phosphorus, and dry skin when daily doses of PD0325901 exceeded 4 mg/kg. In contrast, when the dose of PD0325901 was 3.5 mg/kg, we found no adverse effects over two weeks.

**Figure 2:**
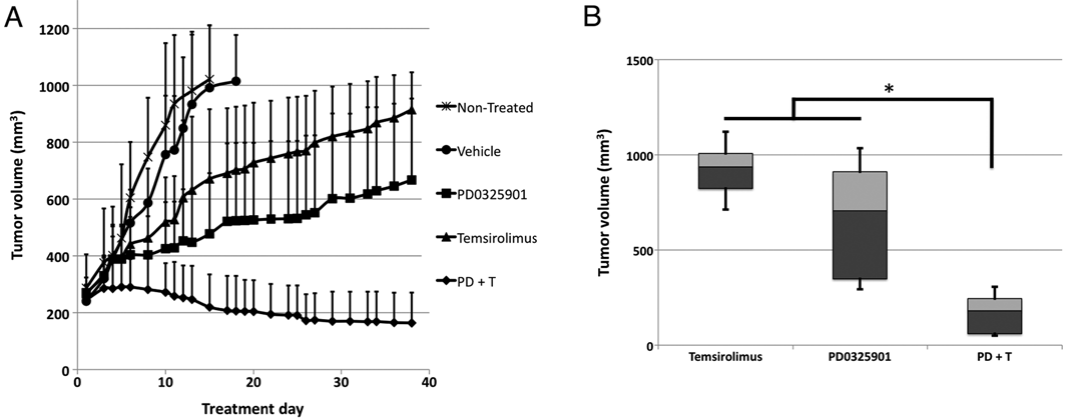
Combined treatment with PD0325901 and temsirolimus inhibits tumor growth. (A) Growth curves of tumorgrafts treated with PD0325901 (3.5 mg/kg) plus temsirolimus (1.87 mg/kg) (n=7), PD0325901 (n=8), temsirolimus (n=8), vehicle (n=8), and non-treated (n=7). (B) At treatment day 38, tumorgrafts treated with PD0325901 plus temsirolimus were significantly smaller than those treated with either PD0325901 or temsirolimus alone. Error bars represent standard deviation. Significance was determined by Wilcoxon rank sum for unmatched pairs. ^*^p<0.005.

Consequently, a 4:1 molar ratio of PD0325901 (at 3.5 mg/kg) and temsirolimus (at 1.9 mg/kg) was used to treat mice bearing canine cardiac angiosarcoma tumorgrafts. After only two weeks, the PD0325901/temsirolimus combination decreased the tumor volume. By three weeks, all vehicle control tumorgrafts had grown to 1000 mm^3^, while tumorgrafts treated with PD0325901/temsirolimus had virtually no growth. On day 38 of treatment, the tumors were significantly smaller than those treated with either PD0325901 or temsirolimus alone (Fig. 2A and B). No weight loss was found in mice treated with the combination over the treatment period. Thus, the combination of MEK and mTOR inhibition produced an efficacious response with no observable toxicities.

**Figure 3:**
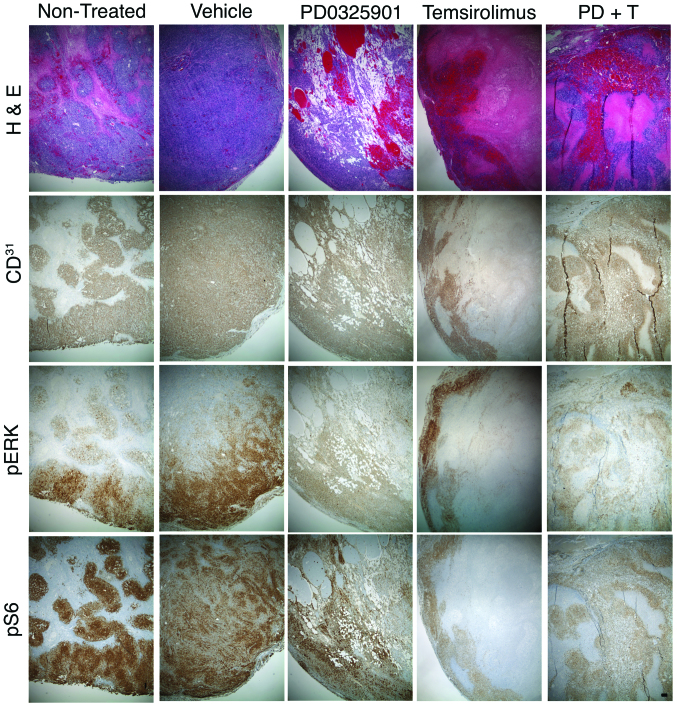
Treatment with PD0325901 and temsirolimus inhibits tumor cell signaling. Subcutaneous tumorgrafts of cardiac canine AS non-treated or treated with vehicle, PD0325901, temsirolimus, or PD0325901 plus temsirolimus (PD+T) were stained for H&E and immunostained for CD^31^, pERK and pS6. Bar, 100 μm.

To determine the morphologic consequences of these treatments, thin sections of formalin-fixed, paraffin-embedded tumors were evaluated by H&E staining. cAS tumorgrafts showed a complex architecture as previously described (8). At the tumor periphery, CD^31+^ cells were arranged within dense tumor nests and lined poorly formed vascular channels. In the tumor interior, large irregular blood vessels were lined with CD^31+^ cells that, in places, were multiple cell layers thick. Such tumors contain large areas of necrosis and fibrin deposition from intratumor infarcts caused by hemorrhage (Fig. 3).

With PD0325901 treatment, these necrotic regions and fibrin deposits were replaced by areas containing small, irregular, perfused vessels lined with CD^31+^ cells. Temsirolimus had the opposite effect, producing a tumor interior that was mostly necrotic and fibrotic, with CD^31+^ cells lining the tumor cortex and few areas of CD^31+^ cells in the tumor interior. The combination therapy showed a mix of these two architectures. There was an increase in small, perfused, CD^31+^ lined vessels, but the necrotic regions were increased relative to PD0325901 treatment alone (Fig. 3). These results indicate that the effects of each drug on tumor architecture were independent of each other.

To determine the molecular consequences of these treatments, we next used immunohistochemistry to examine changes in pERK and pS6 in treated tumorgrafts. PD0325901 reduced pERK staining intensity at the tumor periphery; there was no noticeable decrease in the weak pERK1/2 signal in the tumor interior. PD0325901 did not appear to change pS6 staining intensity. Temsirolimus reduced pS6 staining at the cortex but the residual interior signal was still present. Temsirolimus produced no decrease of pERK staining intensity in viable cells. Tumors from mice treated with PD0325901 plus temsirolimus showed reduced pERK and pS6 staining (Fig. 3). These results indicate that each drug effectively inhibited its intended target, but their combination did not enhance their effects on these targets.

**Figure 4:**
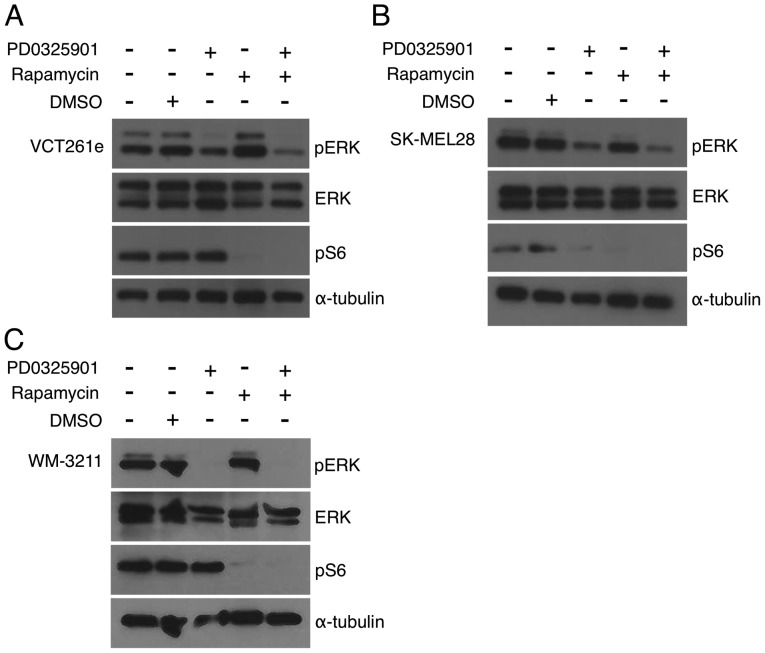
Treatment with PD0325901 and rapamycin inhibits ERK and S6 phosphorylation. (A) VCT261e canine AS primary cell isolates treated with PD0325901 (40 nM) and rapamycin (10 nM) for 72 h. (B) SK-MEL28 cells treated with PD0325901 (5 nM) and rapamycin (6.25 nM) for 72 h. (B) WM-3211 cells treated with PD0325901 (200 nM) and rapamycin (50 nM).

Recent studies of rhabdomyosarcoma have concluded that combined inhibition of MEK and mTOR is synergic because of anti-counteractive interaction: each drug blocks reciprocal activation of the other pathway (31,32). To determine whether the same is true for AS, we performed immunoblots with antibodies against phosphorylated ERK and S6. Canine angiosarcoma primary cell isolates were treated with each drug alone or with a 4:1 (PD0325901:rapamycin) ratio (Fig. 1A). Treatment with 40 nM PD0325901 alone resulted in no effect or minimal reduction of pERK, which is consistent with PD0325901 at this dose having a minimal effect on cell viability. Rapamycin treatment at 100 nM was sufficient to decrease pS6 to nearly undetectable levels in cAS primary isolates (Fig. 4A) and to reduce the pS6 signal to a level consistent with pathway inhibition (Fig. 4A and B). The addition of rapamycin with PD0325901 resulted in only a minor reduction of pERK (Fig. 4A). Similar results were observed in other cAS primary cells (data not shown). SK-Mel28 cells were treated alone and PD0325901 and rapamycin at a 4:5 molar ratio. PD0325901-treated SK-MEL-28 reduced pERK and pS6 levels (Fig. 4B). WM-3211 cells were treated with PD0325901 and rapamycin alone and at a 4:1 molar combination. While PD0325901 and rapamycin were sufficient to reduce pERK and pS6 levels, respectively, combination treated did not further reduce phosphorylation levels (Fig. 4C). While we see no evidence of direct reciprocal activation, these data suggest that mTOR inhibition sensitizes cells to even small reductions in MEK signaling.

### PD0325901 and rapamycin largely results in individual pathway inhibition

**Figure 5:**
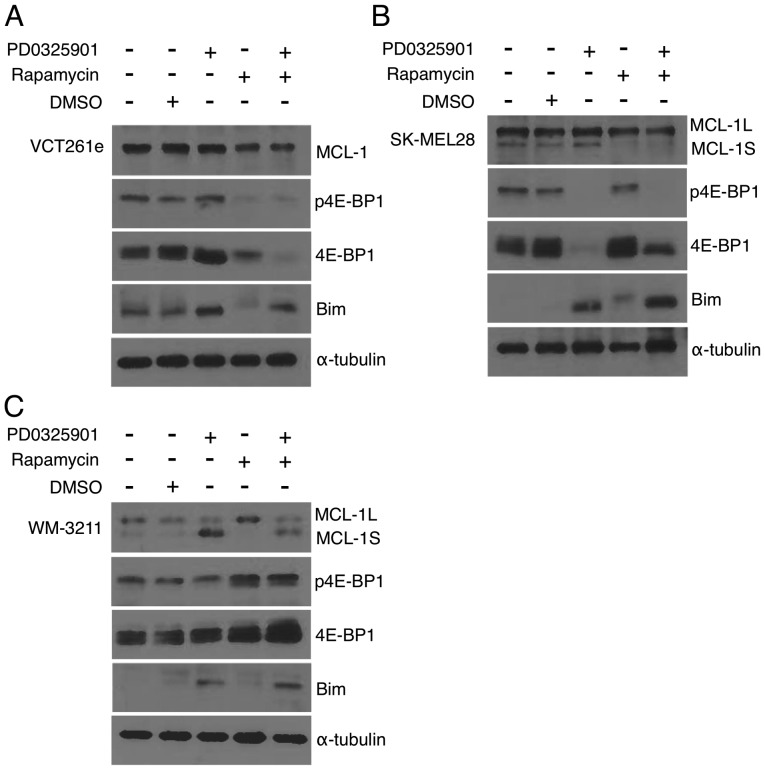
Treatment with PD0325901 and rapamycin alters the expression of pro-survival and pro-apoptotic proteins. MCL-1, phosphorylated 4E-BP1 (S65), total 4E-BP1, Bim, and α-tubulin western blots after dual combination treatment *in vitro*. (A) VCT261e canine AS cells treated with PD0325901 (40 nM) and rapamycin (10 nM) for 72 h. (B) SK-MEL28 cells treated with PD0325901 (5 nM) and rapamycin (6.25 nM) for 72 h. (C) WM-3211 cells treated with PD0325901 (200 nM) and rapamycin (50 nM).

Since combinatorial MEK and mTOR inhibition showed no reciprocal activation or synergistic decrease in ERK and S6 phosphorylation, we next looked at levels of 4E-BP1. Phosphorylation of 4E-BP1 is reported to be regulated through both the MEK and mTOR pathways in MEK driven tumors (33,34). 4E-BP1 inhibits the 5′ mRNA cap recognition of eIF4F complex repressing translation (35). Phosphorylation of 4E-BP1 impedes the binding of 4E-BP1 to eIF4E allowing for eIF4F complex formation and translation initiation (36). For these experiments cells were treated with drugs at the previously determined optimal ratio. For VCT261e cAS primary cell isolates, 4E-BP1 was unaffected by MEK inhibition. In contrast, rapamycin caused a marked reduction in total 4E-BP1 levels (Fig. 5A). This effect was more pronounced in cells treated with both agents. The opposite was observed in SK-MEL-28 cells (Fig. 5B). MEK inhibition reduced 4E-BP1 levels, while rapamycin had no effect. Combined treatment with both drugs caused a partial reduction in 4E-BP1 levels and a loss of phosphorylation. In WM-3211 cells, neither drug markedly altered 4E-BP1 expression or phosphorylation (Fig. 5C).

Pro-survival MCL-1 protein levels were analyzed. MCL-1 is cooperatively regulated by MEK and mTOR in the MEK driven OCM1A melanoma cell line (33), and mTOR is known to induce cell survival through upregulation of MCL-1 protein (37). MCL-1 can exist in two splice variants. MCL-1L is known as a pro-survival protein. MCL-1S is hypothesized to bind and inhibit MCL-1L to block cell survival. Only one splice form was detected in canine cells. In VCT261e cells, expression of MCL-1 was unaffected by PD0325901 and modestly reduced by rapamycin (Fig. 5A). A similar result was achieved in SK-MEL-28 cells where rapamycin reduced levels of both the MCL-1L and MCL-1S (Fig. 5B). However, in WM-3211 cells while PD0325901 alone induced expression of MCL-1S only and rapamycin alone induced the expression of MCL-1L (Fig. 5C), the combination treatment did not have a marked effect.

Finally, we examined the expression of pro-apoptotic Bim. For all three cell types we observed PD0325901, alone or in combination with rapamycin, induced Bim expression (Fig. 5A–C) while rapamycin had no effect. With the exception of 4E-BP1, there appears to be no cooperative modulation of these pathways. In fact, two independent prodeath pathways were present. Rapamycin decreased pro-survival MCL-1, and PD0325901 increased pro-apoptotic Bim expression (Fig. 5A). Collectively, our data indicate in these cell lines that although PD0325901 induces expression of the pro-apoptotic protein Bim and rapamycin reduces levels of the pro-survival protein MCL-1L, there is no evidence that these two inhibitors have a common target.

## Discussion

Since angiosarcomas are rare compared to other cancers, such as breast and lung cancer, they are relatively understudied. Perhaps this is why so few advances have been made in the treatment of angiosarcomas in the past 20 years. Our most effective weapon against angiosarcoma is still a surgeon’s scalpel. Radiation therapy or chemotherapies such as doxorubicin can delay progression, but they cannot prevent it. One reason for our slow progress is the lack of a clear molecular target. Angiosarcoma has no single identifiable genomic cause; mutations in several different genes have been reported. Most recently, Bejhati *et al* reported mutations in the angiogenesis-related genes *PTPRB* and *PLCG1* in 10/39 and 3/34 tumors, respectively (3). In addition, constitutive activation of *KRAS-2* (4–6) and of VEGF receptor-2 (7) has been documented. Of note, several of these signal through the MEK/ERK signaling pathway (38–41).

Recently, we published a study showing that human and canine angiosarcomas express focal to widespread active ERK and are sensitive to MEK inhibition (8). Thus, targeting MEK signaling may be an effective therapy. The goal of this follow-up study was to identify drugs or compounds that synergize with the MEK inhibitor PD0325901 in order to develop a more effective treatment. A recent study highlighting the potential benefits of combination therapies for oncology stated that intratumor heterogeneity, the rapid evolution of bypass mechanisms, and genomic instability lessen the likelihood that monotherapies will provide sustained patient benefit (42). The authors conclude, and we agree, that combination therapy is the future for treating oncology patients.

A recent survey of clinical articles (43) involving drug combinations found that the term synergy is frequently used without an appropriate understanding of either the underlying concept or of the computational approaches to evaluate it, only 20% of preclinical research articles used appropriate methods. This is a concern since the misinterpretation of this concept can adversely impact the formulation of drug combinations in clinical studies. Each of the computational approaches we can use to evaluate drug interactions has its strengths and weaknesses (23,44,45). We chose the methods of Chou and Talalay (23) because they are commonly used to objectively evaluate synergy and because the software needed to make the calculations is freely available. We used cell viability data to calculate the combination index (CI), which is used to objectively evaluate whether two drugs interact in an additive, synergic, or antagonistic fashion. A CI value <1 is considered synergic and a value >1 is considered antagonistic. A CI of 1 is additive.

Using this approach, we have discovered that melanoma and angiosarcoma were insensitive to mTOR inhibition. Treatment with nanomolar levels of mTOR inhibitor, however, rendered these cells as sensitive to MEK inhibition as melanoma with mutant BRAF. This effect was also seen *in vivo*, treatment of tumorgrafts with MEK plus mTOR inhibitors was more effective than monotherapy. Furthermore, MEK-insensitive WM-3211 cells responded to MEK inhibitors when treated simultaneously with nanomolar amounts of rapamycin. This shows that a low dose of an mTOR inhibitor can dramatically enhance the response to MEK inhibition and potentially widen the applications of MEK-targeted therapy.

Combinations of MEK and mTOR inhibitors have been tested in several carcinomas, including lung cancer (46–48), melanoma (49), colorectal cancer (50), and pancreatic cancer (51), but their combined effect on sarcomas have only been reported for rhabdomysarcoma (31,32). MEK 1 and 2 have essential roles in fundamental cellular activities including cell survival, proliferation, motility, and differentiation, as well as in vital activities such as angiogenesis and immune response (52,53). Similarly, the PI3K/AKT/mTOR survival pathway regulates diverse processes such as cell proliferation, differentiation, metabolism, cytoskeletal organization, apoptosis, and cancer-cell survival (54). Mechanistically, two drugs can show synergy because they have anti-counteractive actions, complementary actions, or facilitating actions (55). Because of the diverse functions of the MEK1/2 and the PI3K/AKT/mTOR pathways, it is not clear how inhibitors of each pathway will interact or synergize. Understanding the biologic mechanisms underlying synergy is important to help identify biomarkers of response as well as novel, efficacious combinations.

Recent studies in rhabdomyosarcoma have concluded that combined inhibition of MEK and mTOR is synergic because of anti-counteractive interaction: each drug blocks reciprocal activation of the other pathway (31,32). However, these results are based on *in vitro* effects and do not take into account *in vivo* tumor:stromal interactions. Moreover, the data (immunoblots) are qualitative and cannot be used objectively to determine whether the effects are synergic, additive, or antagonistic. In angiosarcomas we see no convincing evidence by immuno-blotting or immunohistochemistry that either drug promotes activation of the other pathway. Instead, our data indicate that independent pathways are affected. *In vitro*, PD0325901 alone increases the pro-apoptotic protein Bim while treatment with rapamycin decreases pro-survival MCL-1. *In vivo*, PD0325901 results in vascular changes, and temsirolimus affects survival. Based on this, we hypothesize that these pathways signal independently to promote angiosarcoma growth and vascularization.

An alternative possibility is that these signaling pathways converge on a common, not-yet-identified target or activity that is required for angiosarcoma progression. For example, several studies indicate that each of these pathways regulates angiogenesis. Endothelial AKT overexpression increases *in vivo* angiogenesis (56), and rapamycin has been reported to inhibit tumor angiogenesis in xenografts (57). Similarly, constitutive expression of MEK1 in fibroblasts elevates expression of VEGF mRNA through the binding of the transcription factors Sp1 and AP-2 to its promoter region (58). In addition, the treatment of endothelial cells with VEGF causes activation of both ERK 1 and 2 (59). Anthrax lethal factor, a protease that inactivates MEK1 and 2 (60) as well as mitogen-activated protein kinases 3, 4, 6 and 7 (61), substantially inhibits vascularization in mouse xenograft studies (62) and in models of retinal angiogenesis (63,64). Thus, the PI3K/AKT/mTOR and MEK/ERK signaling pathways may converge on an angiogenesis-related target required for angiosarcoma progression.

## Figures and Tables

**Table I:** Calculated IC_50_ for a single dose of PD0325901, rapamycin, or both at the optimal molar ratios for cAS primary cell isolate VCT261e and the melanoma cell lines SK-MEL28 and WM-3211.

	cAS	Melanoma	
		
Treatment	VCT261e	SK-MEL28	WM-3211
PD0325901			
IC_50_ (nM)	150±30	20±4	>1,000
Rapamycin			
IC_50_ (nM)	>50	7±11	>50
PD0325901 + Rapamycin			
IC_50_ (nM)	11±6	6±8	250±250
CI	0.07	0.07	0.0003
Molar ratio	4:1	4:5	4:1

CI, combinatorial index.
